# Cost-effectiveness of the ABC_Stroke_ pathway in ischaemic stroke care: a UK pilot analysis

**DOI:** 10.1093/ehjqcco/qcaf157

**Published:** 2026-01-22

**Authors:** Borislav Borissov, Dimitrios Sagris, Jacopo Imberti, Anna Podlasek, Mondher Toumi, Renate B Schnabel, Dimitrios N Nikas, George Ntaios, Konstantinos Vemmos, Nikola Kozhuharov, Gregory Y H Lip, the ESC Council on Stroke collaboration, Alison Halliday, Marco Proietti, Giulio F Romiti, Giuseppe Boriani, Milos Taborsky, Petr Widimsky

**Affiliations:** Faculty of Public Health, Medical University of Sofia, Boulevard "Akademik Ivan Evstratiev Geshov " 15, 1431 Sofia, Bulgaria; Liverpool Centre for Cardiovascular Science at University of Liverpool, Liverpool John Moores University and Liverpool Heart & Chest Hospital, Liverpool, UK; Department of Internal Medicine, General University Hospital of Larissa, Faculty of Medicine, School of Health Sciences, University of Thessaly, Gaiopolis Campus, Larissa-Trikala Ring Road, Larisa 413 34, Greece; Liverpool Centre for Cardiovascular Science at University of Liverpool, Liverpool John Moores University and Liverpool Heart & Chest Hospital, Liverpool, UK; Department of Biomedical, Metabolic and Neural Sciences, University of Modena and Reggio Emilia, and Azienda Ospedaliero-Universitaria di Modena, Science Campus Biomedical Sciences Building via Giuseppe Campi, 287 41125 Modena, Italy; Image Guided Therapy Research Facility (IGTRF), University of Dundee, Wilson House, 1 Wurzburg Loan, Dundee Medipark, Dundee DD2 1FD, UK; Department of Public Health, Aix-Marseille University, 51 Pierre Dramard boulevard, 13344 Marseille, France; Department of General and Interventional Cardiology, University Heart & Vascular Center Hamburg, University Medical Center Hamburg-Eppendorf, Martinistrasse 52, 20246 Hamburg, Germany; First Cardiology Department, Ioannina University Hospital, Stavrou Niarchou Avenue, 45500 Ioannina, Greece; First Propaedeutic Department of Internal Medicine, AHEPA University Hospital, Aristotle University of Thessaloniki, Kiriakidi 1, Thessaloniki 546 36, Greece; Hellenic Cardiovascular Research Society, 6 Potamianou, 115 28 Athens, Greece; Department of Cardiology, University Hospital Bern, Freiburgstrasse 15, 3010 Bern, Switzerland; Cardiovascular Research Institute Basel, Spitalstrasse 2, 4056 Basel, Switzerland; Liverpool Centre for Cardiovascular Science at University of Liverpool, Liverpool John Moores University and Liverpool Heart & Chest Hospital, Liverpool, UK; Danish Center for Health Services Research, Department of Clinical Medicine, Aalborg University, Selma Lagerløfs Vej 249 (Building 11, 3rd floor), 9260 Aalborg, Denmark; Department of Cardiology, Lipidology and Internal Medicine with Intensive Coronary Care Unit, Medical University of Bialystok, ul. Żurawia 14, 15-540 Bialystok, Poland

**Keywords:** ABC_Stroke_ pathway, Cost-effectiveness, Ischaemic stroke, Secondary prevention, Health economics, Markov model

## Abstract

**Aims:**

The ABC_Stroke_ pathway is a structured, person-centred approach to post-stroke care encompassing antithrombotic therapy, functional and psychological optimization, and cardiovascular risk factor management, proposed by the European Society of Cardiology Council on Stroke. Adherence to the pathway has been associated with improved outcomes in observational studies, but its cost-effectiveness remains unknown. We evaluated the long-term cost-effectiveness of the ABC_Stroke_ pathway in the UK.

**Methods and results:**

A Markov model was developed to simulate lifetime costs and outcomes of patients following an ischaemic stroke, using clinical data from the Athens Stroke Registry and UK-specific cost and utility inputs from the Oxford Vascular Study. Patients were stratified by adherence to all three ABC_Stroke_ pathway components vs. non-adherence. Over a 20-year horizon, pathway-aligned/adherent care was associated with higher costs (£129 143 vs. £71 472) and greater quality-adjusted life years (QALYs; 9.84 vs. 5.94), yielding an incremental cost-effectiveness ratio (ICER) of £14 804 per QALY gained. At a willingness-to-pay threshold of £25 000 per QALY, the net monetary benefit of the ABC_Stroke_ pathway was £116,857, compared with £77 028 for non-aligned care, indicating a net gain of £39 829 in favour of the ABC-aligned strategy. Deterministic sensitivity analyses showed the ICER remained below £16 000 per QALY across plausible parameter ranges. Even assuming a 100-fold increase in pathway implementation costs, the strategy remained within or near the lower boundary of the UK National Institute for Health and Care Excellence willingness-to-pay threshold.

**Conclusion:**

In the UK, implementation of the ABC_Stroke_ pathway is cost-effective. These findings support strategic investment in structured, multidisciplinary integrated care for stroke survivors.

## Graphical Abstract

**Graphical Abstract:**
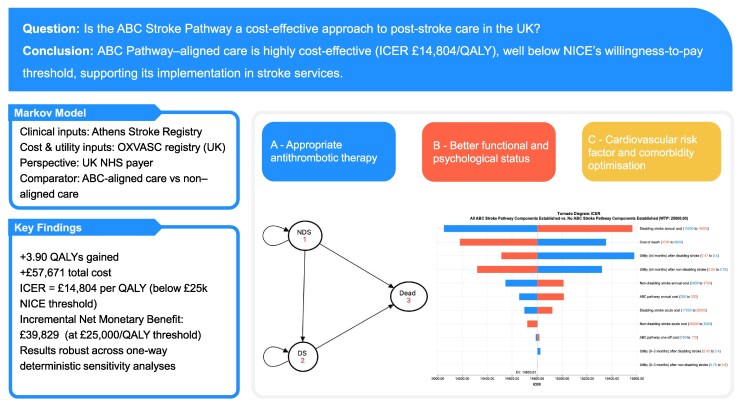
Cost-effectiveness of the ABC Stroke Pathway in the UK. ICER—Incremental cost-effectiveness ratio; NDS—non-disabling stroke (modified Rankin Scale 0–2); DS—disabling stroke (modified Rankin Scale 3–5).

Key Learning Points What is already known:

- The ABC_Stroke_ pathway is a structured approach to post-stroke care encompassing antithrombotic treatment, functional and psychological recovery, and cardiovascular risk factor management.
- Observational data from European and Asian cohorts suggest that adherence to the ABC_Stroke_ pathway is associated with lower rates of recurrent stroke, major adverse cardiovascular events, and all-cause mortality.
- Despite growing evidence of its clinical benefit, economic data supporting broader adoption of the pathway have been lacking.

What this study adds:

- Using a Markov model, this study demonstrates that full adherence to the ABC_Stroke_ pathway is cost-effective in the UK, yielding an incremental cost-effectiveness ratio of £14 804 per quality-adjusted life year—well within National Institute for Health and Care Excellence thresholds.
- The findings remain robust across a wide range of assumptions, with the pathway retaining cost-effectiveness even under exaggerated implementation cost scenarios.
- These results support strategic investment in pathway-aligned, multidisciplinary secondary prevention approaches to post-stroke care in the NHS setting.

## Introduction

Stroke remains a leading cause of death and disability in the United Kingdom, with estimated total costs exceeding £26 billion, including hospital care, long-term support, and productivity losses.^1^ While acute heart failure is often cited as the leading cause of unplanned hospital admissions in high-income countries,^2-4^ in the UK, stroke now surpasses heart failure, accounting for 142 641 hospitalizations in 2024 as compared with 119 764 for heart failure, reflecting its rising incidence and substantial burden on inpatient care.^5,6^

Despite improvements in acute stroke management, long-term secondary prevention remains fragmented.^7^ Many patients are discharged without a systematic plan for secondary prevention, functional recovery, or long-term management of cardiovascular risk factors, resulting in missed opportunities to reduce recurrent stroke and hospitalization.^7^ This is important given the high cardiovascular risks associated with the post-stroke patient and the presence of the stroke-heart syndrome whereby ∼20–25% of post-stroke patients are at risk of incident cardiovascular events.^8-10^

In 2022, the European Society of Cardiology Council on Stroke proposed the use of the ABC_Stroke_ pathway as a holistic and integrated care approach to post-stroke care.^7,11^ The ABC_Stroke_ pathway reflects three key domains: (i) Appropriate antithrombotic therapy; (ii) Better functional and psychological status, supported by early rehabilitation and systematic screening for cognitive impairment and depression; (iii) Cardiovascular risk factor and comorbidity optimization, including blood pressure and lipid management, glycaemic control, and lifestyle interventions.

Real-world data from the Athens Stroke Registry have demonstrated that full adherence to the ABC_Stroke_ pathway is associated with significant reductions in all-cause mortality, recurrent stroke, and major adverse cardiovascular events.^12^ These findings have been independently replicated in a cohort from Hong Kong, reinforcing the consistency of its clinical benefit across diverse healthcare systems.^13^

While its clinical effectiveness is now suggested by real-world observational data, the cost-effectiveness of the ABC_Stroke_ pathway has not been evaluated. This study addresses this gap in knowledge by modelling the long-term economic and health outcomes associated with the implementation of the ABC_Stroke_ pathway in the UK stroke population.

## Methods

### Model structure

**Figure 1:**
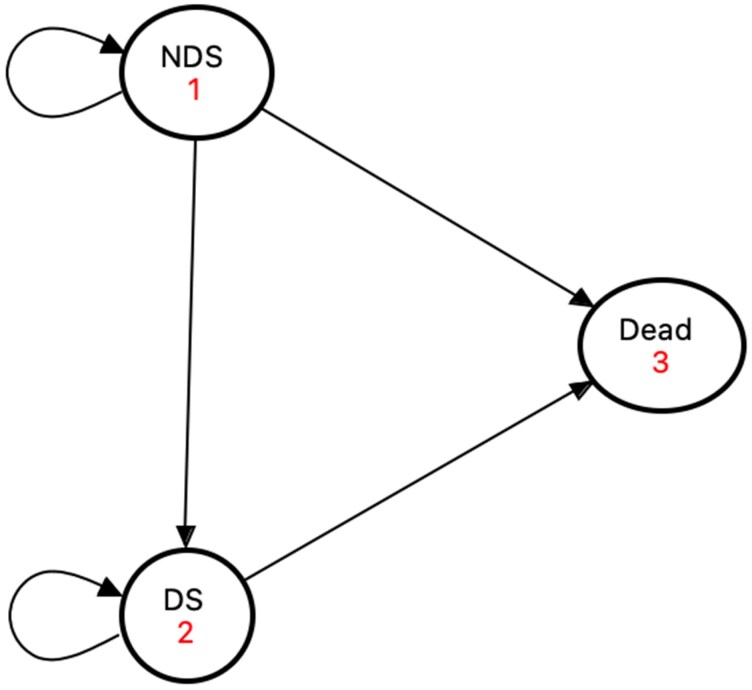
State transition diagram of the Markov model used to evaluate the cost-effectiveness of the ABC stroke pathway. Patients enter the model after an index ischaemic stroke in either a NDS or a disabling stroke health state and can transition annually between health states or to death. Recurrent strokes are accounted for within the transition probabilities. For instance, NDS (modified Rankin Scale 0–2) patients may remain in the same health state after a recurrent NDS or progress from NDS to DS (modified Rankin Scale 3–5) following a disabling recurrent stroke. .

We developed a Markov model to estimate the long-term cost-effectiveness of the ABC_Stroke_ pathway compared with care not aligned with the pathway. The model transition diagram and detailed structure are illustrated in *Figure 1* and Supplementary material online, *Figure S1*.

Patients entered the model after an index ischaemic stroke and were stratified into two health states based on their post-stroke functional status at baseline: non-disabling stroke [NDS; modified Rankin Scale (mRS) 0–2] and disabling stroke (DS; mRS 3–5).^10,11^ This stratification reflects clinically meaningful differences in health-related quality of life, healthcare resource utilisation, and stroke outcomes.^14,15^

Each annual cycle, patients could remain in their current health state, experience a recurrent ischaemic stroke (either with or without a functional progression), or die. Transitions from NDS to DS following a recurrent stroke were modelled explicitly, while patients with DS could only remain in DS or transition to death. This assumption reflects that the model starts at 3 months post-stroke, when most functional recovery has already occurred.^16^ Although recovery may continue after 3 months, detailed longitudinal mRS changes were not consistently captured. To partly address this, patients were grouped into broader categories of disabling (mRS 3–5) and non-disabling (mRS 0–2) stroke. The model therefore uses the 3-month functional status as its starting point and does not incorporate further recovery, as these data were not consistently available in the registry. The model was developed in TreeAge Pro 2025, R2.0 (TreeAge Software, Williamstown, MA), and all analyses were conducted using this software.

### Clinical inputs

Transition probabilities were derived from individual patient-level data from the Athens Stroke Registry, a prospective cohort of consecutive patients with ischaemic stroke admitted to Alexandra University Hospital, Athens, Greece, between June 1992 and December 2011. The scientific use of the data collected in the Athens Stroke Registry was approved by the local Ethics Committee. All data in the registry were prospectively collected, including demographics, medical history, and associated cardiovascular risk factors, current medication, time of stroke onset and hospital admission, duration of hospitalization, stroke characteristics, clinical findings and vital signs on admission, laboratory investigations, and treatment. Stroke severity was assessed by means of the National Institute Health Stroke Scale score at admission.^17,18^ Patients were stratified according to adherence to the ABC_Stroke_ pathway, based on available data from the Athens Stroke Registry as follows:

‘**A’ Criterion: Appropriate Antithrombotic therapy**. Stroke patients were considered adherent to the ‘A’ criterion if they have been prescribed the appropriate antithrombotic treatment after stroke at discharge and after stroke aetiology work-up. Thus, patients with AF or prosthetic valve were adherent to ‘A’ if they have been prescribed at baseline with OAC according to the baseline thromboembolic risk, whereas stroke patients without AF or any other reason for anticoagulation were adherent if they had been prescribed with antiplatelet therapy. All other patients were considered non-adherent.

‘**B’ Criterion:** Better functional and psychological status. To be adherent with the ‘B’ criterion, all stroke patients with any deficit at discharge, defined based on the mRS ≥ 1, should have been prescribed with any kind of physiotherapy, speech therapy, or ergotherapy, irrespective of the intensity of the therapy.

‘**C’ criterion:** Cardiovascular risk factors and comorbidity optimization. For the ‘C’ criterion in stroke patients, we considered the use of statin, treatment of hypertension if the patient was diagnosed with hypertension and hypoglycaemic treatment if the patient was diagnosed with diabetes mellitus, based on the characteristics of the patients. Additionally, in case of carotid stenosis ≥70% ipsilateral to the index stroke, adherence to the ‘C’ criterion was met when the patient was treated with carotid revascularization. Only patients with available data on baseline stroke severity (mRS at three months) were included in the present analysis. Separate transition probabilities were calculated for the full adherence and no adherence groups based on observed rates of stroke recurrence, all-cause mortality, and functional decline during follow-up.

### Utilities

Health-related quality of life estimates were derived from patients with ischaemic stroke in the Oxford Vascular Study (OXVASC), which provides UK-specific EQ-5D utility values mapped to mRS categories.^19,20^ We applied a utility of 0.80 for patients with NDS and 0.45 for those with DS, based on values reported by Rivero-Arias *et al.*^19^ Utility inputs are detailed in Supplementary material online, *Table S1* and are consistent with those used in prior stroke economic evaluations, including Tank *et al.*^14^ Utilities were assumed to remain constant over time unless a recurrent stroke resulted in a change in functional status. For instance, the same utility values were applied for initial and recurrent NDSs due to the lack of separate estimates in the OXVASC dataset.

### Costs

Cost inputs were taken from the OXVASC study in AF-related stroke,^20-22^ which provided event-severity stratified estimates suitable for the structure of our Markov model.^21,22^ Cost categories included index hospitalization and post-acute care, long-term care, recurrent stroke events, and end-of-life care. In line with the OXVASC cost analysis by Luengo-Fernandez *et al.* (2013), the cost of death in our model corresponded to the reported cost for case-fatal strokes (defined as deaths within 30 days of the event). The same cost values were applied for initial and recurrent strokes due to the absence of separate estimates in the OXVASC dataset. ABC_Stroke_ pathway delivery costs were derived from NHS reference costs and weighted by the prevalence of comorbidities in the Athens Stroke Registry,^12,22^ reflecting the proportion of patients requiring specific interventions within each component, e.g. for prevalence of hypertension and need for antihypertensive treatment under the ‘C’ domain (cardiovascular risk/comorbidity optimization). Full details of all cost inputs are provided in Supplementary material online, *Tables S2* and *S7*. For the single-component adherence sensitivity analysis, costs were estimated as one-third of those for full ABC_Stroke_ pathway implementation, reflecting proportional resource use. Baseline comorbidities were derived from the Athens Stroke Registry, and their prevalence is presented in Supplementary material online, *Table S10,* for comparison with published OXVASC data.

### Perspective and time horizon

The analysis was conducted from the perspective of the UK. Costs were estimated from the NHS and social services payer perspective. A 20-year time horizon was adopted. Future costs and quality-adjusted life year (QALYs) were discounted at an annual rate of 3.5%, in line with UK’s National Institute for Health and Care Excellence (NICE) guidance.^23^

### Sensitivity analyses

Deterministic sensitivity analyses were conducted on key input parameters, including utilities and costs, to assess the robustness of the results. Probabilistic sensitivity analysis (PSA) was performed in TreeAge Pro using Monte Carlo simulation with 10 000 iterations. Transition probabilities derived from the Athens Stroke Registry were modelled as beta distributions. PSA for utilities and costs was not undertaken due to unavailable variance parameters. To mitigate small-sample and boundary instabilities in transition probabilities, we applied Agresti–Coull and Wilson adjustments at 95% confidence. Adjusted estimates were used in probabilistic analyses, respectively. An additional comparison was conducted between patients with any single component of the ABC_Stroke_ pathway established and those with full adherence (all three components). All analyses were conducted in R within a Jupyter notebook on Julius (https://julius.ai) and TreeAge Pro 2025, R2.0.

Willingness-to-pay thresholds were set at £25 000 per QALY gained, consistent with NICE guidance (20 000 to £30 000) for cost-effectiveness analyses in the UK.^23-26^

## Results

### Athens stroke registry cohort

Clinical event rates were derived from the Athens Stroke Registry, which prospectively enrolled consecutive patients with ischaemic stroke between 1992 and 2012. The median age at baseline was 71 years (IQR 62–78), and 37.7% were female.

Of 2513 patients, 156 (6.2%) received care aligned with all three ABC_Stroke_ pathway components, and 192 (7.6%) received care aligned with none. The remaining patients had partial adherence, accordingly. Detailed data regarding the baseline characteristics of the included patients based on ABC_Stroke_ can be found in Supplementary material online, *Table S10*. For this analysis, only those with complete 3-month mRS data and either full (*n* = 155) or no adherence (*n* = 152) were included. In the full adherence group, 104 patients (67.1%) had NDS (mRS 0–2) and 51 (32.9%) had DS (mRS 3–5); in the no adherence group, 113 (74.3%) had NDS and 39 (25.7%) had DS. Transition probabilities for recurrent stroke, functional decline, and death were derived from observed outcomes and are presented in Supplementary material online, *Tables S3–S6*. *Table 1* summarises the model inputs, including costs, utilities, and transition probabilities used in the Markov model.

**Table 1:** Health state costs and utilities used in the Markov model

Health state	ABC arm init cost (£)	ABC arm incr cost (£)	ABC arm init utility	ABC arm incr utility	Usual care init cost (£)	Usual care incr cost (£)	Usual care init utility	Usual care incr utility
Non-disabling stroke	£7504.57 = 3743.28 + 133.10 + 3628.19	£3628.19 = 3387.15 + 241.04	0.80	0.80	£7130.43 = 3743.28 + 3387.15	£3387.15	0.80	0.80
Disabling stroke	£34 526.02 = 18 406.92 + 133.10 + 15 986.00	£15 986.00 = 15 744.96 + 241.04	0.45	0.45	£34 151.88 = 18 406.92 + 15 744.96	£15 744.96	0.45	0.45
Recurrent NDS	£3876.38 = 3743.28 + 133.10	—	0.80	0.80	£3743.28	—	0.80	0.80
Recurrent DS after previous DS	£18 540.02 = 18 406.92 + 133.10	—	0.45	0.45	£18 406.92	—	0.45	0.45
Recurrent DS after previous NDS	£18 540.02 = 18 406.92 + 133.10	—	0.45	0.45	£18 406.92	—	0.45	0.45
Dead	£3328	£3328	0	0	£3328	£3328	0	0

Reference values:

cABC pathway delivery initial costs = £133.10.

cABC pathway delivery annual costs = £241.04.

NDS—non-disabling stroke (mRS 0–2); DS—disabling stroke (mRS 3–5); Init—initial (0–3 months); Incr—incremental (≥4 months).

### Base-case analysis

Over a 20-year time horizon, care aligned with the ABC_Stroke_ pathway was associated with higher total costs and greater health benefits compared to care not aligned with the pathway. Specifically, average per-patient costs were £129 143 in the ABC_Stroke_ pathway aligned/adherent group vs. £71 472 in the non-ABC aligned group, with corresponding QALYs of 9.84 and 5.94, respectively.

The resulting incremental cost was £57,671, with an incremental effectiveness gain of 3.90 QALYs, yielding an incremental cost-effectiveness ratio (ICER) of £14 804 per QALY gained. This is well below the commonly accepted UK threshold of £20 000–£30 000 per QALY.

### Net monetary benefit

At a willingness-to-pay threshold of £25 000 per QALY, the net monetary benefit for the ABC_Stroke_ pathway was £116,748, compared with £77 026 for care not aligned with the pathway, indicating an incremental net monetary benefit of £39 722 in favour of the ABC_Stroke_ pathway-aligned strategy.

### Sensitivity analyses

PSA was performed using Monte Carlo simulation with 10 000 iterations, incorporating uncertainty in transition probabilities. The PSA confirmed the robustness of the base-case results, with the majority of simulations falling below the cost-effectiveness threshold. At a willingness-to-pay threshold of £25 000 per QALY, the ABCStroke pathway remained cost-effective in nearly all simulations, and results were consistent even at a lower threshold of £15 000 (see Supplementary material online, *Figure S3A*–3*B*). Net monetary benefit distributions further supported these findings, showing higher mean values for ABCStroke-aligned care compared with non-aligned care (see Supplementary material online, *Figure S4*).

In the comparison of any single ABC_Stroke_ component vs. full adherence, full adherence remained consistently cost-effective. The PSA confirmed higher net monetary benefit and favourable cost-effectiveness across willingness-to-pay thresholds of £15 000 and £25 000 per QALY (see Supplementary material online, *Figures S5* and *S6*).

**Figure 2:**
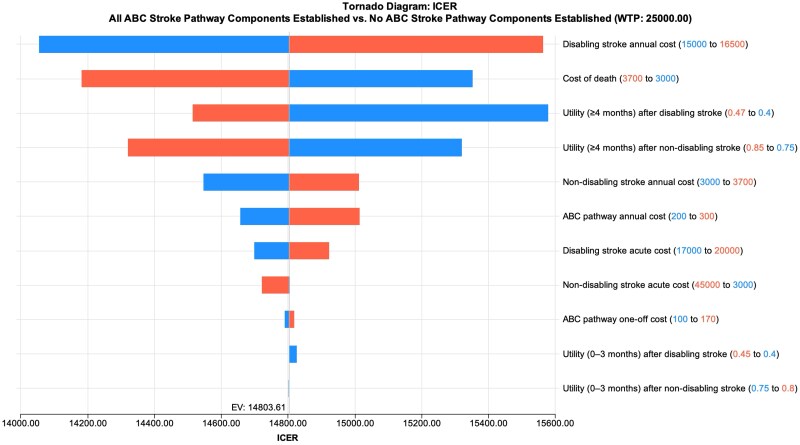
Tornado diagram of one-way sensitivity analyses on ICER for the ABC stroke pathway vs. usual care.

**Figure 3:**
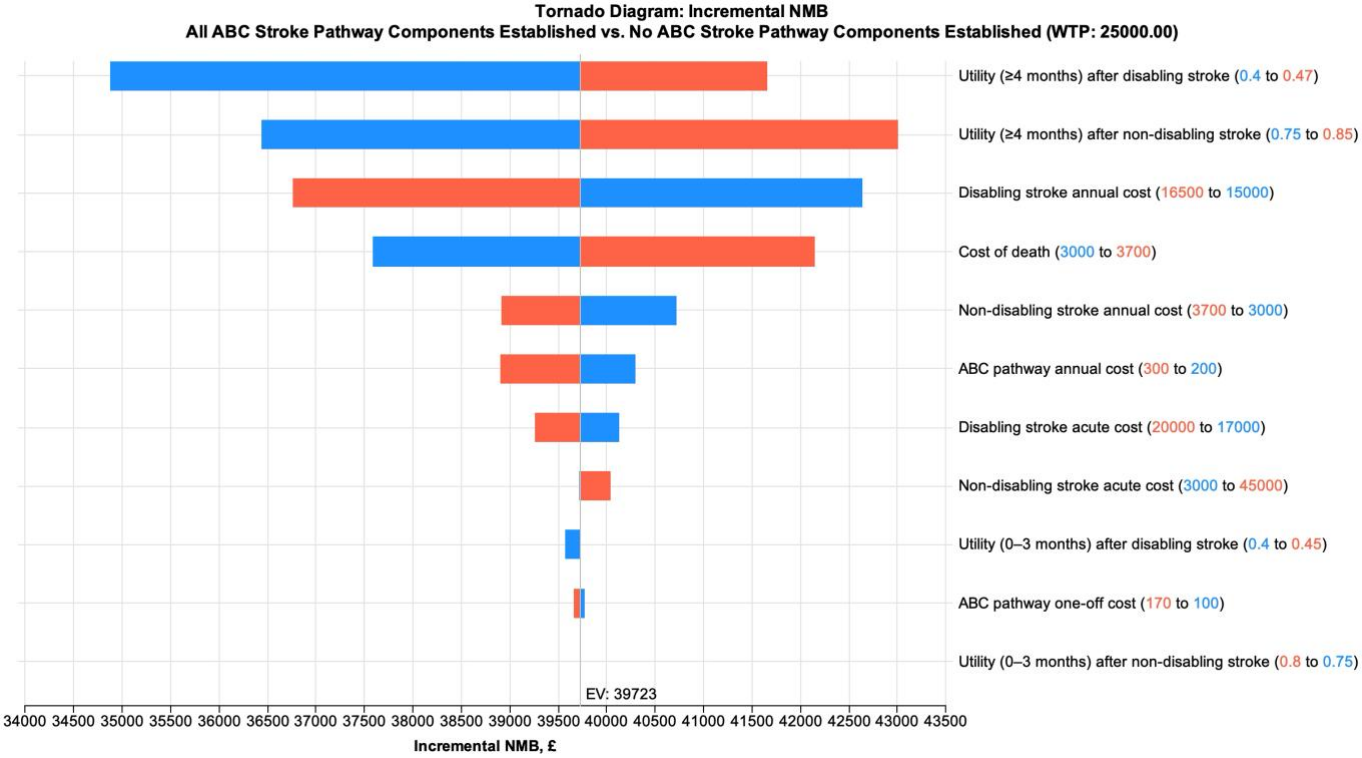
Tornado diagram of one-way sensitivity analyses on incremental net monetary benefit for the ABC stroke pathway vs. usual care at a £25 000/QALY threshold.

Deterministic one-way sensitivity analyses showed that the ICER remained below £16 000 per QALY across plausible variations in key parameters, including acute care costs, utilities, and annual post-stroke costs. The model was most sensitive to long-term care costs and annual utility values following a DS (*Figure 2*). The incremental net monetary benefit consistently exceeded £34,000, with the greatest impact observed for assumptions related to long-term utility and annual costs following DS (*Figure 3*).

Notably, even an excessive (100-fold) theoretical increase in the one-off costs associated with ABC_Stroke_ pathway implementation (for example, to support broader adoption or enhanced infrastructure) resulted in ICERs only slightly exceeding £20 000 per QALY (see Supplementary material online, *Figure S2*), remaining close to the lower end of NHS cost-effectiveness thresholds. In all tested scenarios, the ABC_Stroke_ pathway remained cost-effective.

## Discussion

The principal finding of our study is the demonstration that alignment of stroke care with the ABC_Stroke_ pathway is cost-effective within the UK NHS healthcare context. Our sensitivity analyses confirmed the robustness of the base-case findings across a range of scenarios.

Based on a 20-year Markov model using clinical outcome data from the Athens Stroke Registry and UK-specific cost and utility estimates from the OXVASC study, we show that the ABC_Stroke_ pathway was associated with greater health benefits (+3.90 QALYs) and higher costs (+£57 671), resulting in an ICER of £14 804 per QALY gained. These findings fall well within the accepted UK willingness-to-pay thresholds and support the economic viability of pathway-aligned post-stroke care.^23,25,26^ Notably, the ICER is also below the more conservative £15 000 per QALY threshold proposed by some researchers as a closer estimate of the NHS’s true opportunity cost, further reinforcing the robustness of its value proposition.^26^

The Markov model was designed to reflect the long-term consequences of ischaemic stroke in a stroke cohort and incorporated real-world event rates, stratified by adherence to the ABC_Stroke_ pathway. The holistic and integrated care approach based on the ABC_Stroke_ pathway framework has previously been shown to be associated with clinical benefits in reducing mortality, recurrent stroke, and major adverse cardiovascular events.^12,13^ These outcomes were first reported in the Athens Stroke Registry and independently confirmed in a validation cohort from Hong Kong, underscoring the reproducibility of its effectiveness across healthcare systems. Ongoing clinical trials are in planning stages to formally compare such a strategy to current stroke unit-based ‘usual care’.

Building on this evidence base, our cost-effectiveness model incorporated UK health utility weights stratified by mRS category and healthcare resource use estimates from the OXVASC registry.^19-21^ The analysis confirmed that the ABC_Stroke_ pathway’s clinical benefits translate into long-term economic value, particularly through improved functional outcomes and prevention of stroke recurrence.

One-way sensitivity analyses confirmed the robustness of the base-case findings. The model remained cost-effective across all tested scenarios, with the ICER staying below £16 000 per QALY across plausible variations in utilities, event rates, and costs. Even under extreme assumptions—such as a 100-fold increase in pathway implementation costs—the ICER remained near or within the lower range of NICE’s cost-effectiveness threshold (range £20 000–30 000).^23,25,26^ This resilience highlights the favourable return on investment of structured, evidence-based secondary stroke prevention.

In the UK, while the ABC_Stroke_ framework is not referenced as a single pathway in national guidance, its components are embedded across NICE recommendations. Specifically, antithrombotic management is addressed in NG196 and NG128, structured rehabilitation with cognitive and mood assessment in NG236, and cardiovascular risk-factor optimization in NG238.^27-31^ Nonetheless, national audit data indicate incomplete real-world delivery (e.g. only 39% of eligible patients received a 6-month review in 2023/24),^32^ underscoring the potential health system value of improving pathway-level adherence.

Despite its strong cost-effectiveness profile, real-world implementation of the ABC_Stroke_ pathway remains suboptimal, also outside the UK. Data from both the Athens and Hong Kong cohorts demonstrate that full adherence to all ABC components according to the current guidelines is rare (6.2% and 58.1%, respectively), highlighting a persistent gap between evidence and practice.^12,13^ Behavioural barriers may contribute to this underuse, as observed in other cardiovascular care settings.^33^ Insights from behavioural health economics suggest that addressing these barriers through approaches such as choice architecture, social incentives, and targeted education could increase uptake.^33,34^ Notably, even if implementation costs were to rise substantially (up to 100-fold), the ABC_Stroke_ pathway would remain cost-effective at or near the lower end of NICE’s willingness to pay threshold. These findings support not only the value of structured secondary prevention but also the case for strategic investment to promote its broader adoption. Of note, our findings in the post-stroke care setting reflect cost-effectiveness and modelling projections of adherence with an integrated care management approach in other high-risk chronic cardiovascular conditions, such as atrial fibrillation.^35,36^

### Limitations

Several limitations warrant consideration. The low adherence rate identified in the Athens Stroke Registry is explained by its long duration (starting in 1992), which incorporates all previous stages of evolution of stroke-related medical knowledge and corresponding guidelines, which would be considered inadequate by contemporary medical standards. Transition probabilities were derived from a Greek population but applied to a UK setting with UK-specific cost and utility inputs. However, the ABC_Stroke_ pathway effectiveness has been independently confirmed in an Asian cohort, supporting wider applicability. Further, comorbidity prevalence was taken from the Athens Stroke registry cohort, which shows a slightly higher vascular-risk burden than UK population-based data. This may affect absolute cost estimates but is unlikely to alter overall cost-effectiveness conclusions.^12,37^ Adherence to ABC_Stroke_ pathway was based on the treatment of patients at discharge and at least after the stroke work-up based on the judgement of the treating physician. We do not have robust data on compliance and whether the targets of secondary cardiovascular risk factor management were reached, thus we cannot exclude the possibility that several changes during the follow-up may have potentially influenced the results of our study. Nevertheless, even after taking into consideration this limitation, the adherence to an integrated stroke pathway was found to be associated with a lower risk of stroke recurrence and cardiovascular outcomes.^12^ This work represents a pilot cost-effectiveness analysis within the UK context, as existing registry publications (e.g. OXVASC, SSNAP, Scottish Stroke Care Audit) generally report outcomes only up to the first event or short-term follow-up, limiting their direct use in transition modelling. Future research should address this by incorporating patient-level data from these UK registries, for which the present analysis provides the methodological foundation. Cost estimates were based on AF-related stroke data from OXVASC, which may modestly overestimate costs in a broader stroke population. Notably, the present pilot cost-effectiveness analysis establishes a basis for future validation using patient-level UK registry data. Although all patients were followed yearly for up to 10 years or until death, there might have been changes in the mRS or deaths occurring before mRS re-assessment, which may have introduced bias. We acknowledge that a small proportion of patients with DS may have experienced recovery to a non-disabling state over longer follow-up, which our model does not capture. This simplification was chosen to reflect long-term stability of functional status beyond the early recovery phase. Health-related quality of life and cost data were not collected directly and were mapped from the UK OXVASC registry, which precluded probabilistic sensitivity analyses. Instead, extensive deterministic analyses were conducted for both cost inputs and transition probabilities to test robustness. Costs were assessed from the NHS and personal social services payer perspective and did not include a broader societal perspective, such as productivity losses or unpaid informal care. Separate utility or cost estimates for initial and recurrent strokes were not available, and the same values were applied for both. While some studies have reported no differences in costs or utilities between initial and recurrent strokes,^38^ the evidence remains limited.^39^ Deterministic sensitivity analyses across a wide range of plausible cost and utility values for initial and recurrent strokes confirmed the robustness of the findings. Transition probabilities were derived from a limited sample without baseline adjustment, which may reduce precision. However, this was partly addressed through PSA on transition parameters, including mortality, confirming robustness of findings.

### Conclusion

In this first economic evaluation, the ABC_Stroke_ pathway yielded an ICER of £14 804 per QALY, well within accepted UK thresholds, confirming its cost-effectiveness for post-stroke care. The findings support its broader adoption in stroke services and offer policymakers an evidence-based rationale for investing in structured, multidisciplinary secondary prevention strategies tailored to stroke survivors.

## Collaborators

Additional ESC Council on Stroke Nucleus collaborators# and contributors:

Alison Halliday, MD^1^; Marco Proietti, MD^2^; Giulio F. Romiti, MD^3^; Giuseppe Boriani, MD^4^; Milos Taborsky, MD^5^; Petr Widimsky, MD^6^.

^1^ Medical Research Council Population Health Research Unit at the University of Oxford and Clinical Trial Service Unit, Nuffield Department of Population Health, University of Oxford, Oxford, UK.

^2^ Department of Clinical Sciences and Community Health, University of Milan, Milan, Italy.

^3^ Department of Translational and Precision Medicine, Sapienza University of Rome, Rome, Italy.

^4^ Department of Biomedical, Metabolic and Neural Sciences, University of Modena and Reggio Emilia, and Azienda Ospedaliero-Universitaria di Modena, Modena, Italy

^5^ Cardiology Clinic, Masaryk Hospital, Krajska zdravotni, a.s., Usti nad Labem, Czech Republic.

^6^ Department of Cardiology, Third Faculty of Medicine, Charles University and University Hospital Královské Vinohrady, Prague, Czech Republic.

## Supplementary Material

qcaf157_Supplementary_Data

## Data Availability

The data underlying this article will be shared on reasonable request to the corresponding author.
