# Supplementary material for: Cost-effectiveness of the ABCStroke pathway in ischaemic stroke care: a UK pilot analysis
Source: Eur Heart J Qual Care Clin Outcomes. 2026 Jan 22;12(5):825–32. doi: 10.1093/ehjqcco/qcaf157 (PMC13458152; doi:10.1093/ehjqcco/qcaf157)
Supplement: Supplementary file 1 [file qcaf157_supplementary_data.docx]

## **Supplemental Materials**

### **Supplemental Figure 1. Detailed Markov model structure used for cost-effectiveness analysis of the** ABC_Stroke_ pathway**.**

This figure illustrates the structure of the Markov model comparing care aligned with the ABC_Stroke_ pathway to non-aligned usual care. The model simulates patient transitions among three health states - Non-Disabling Stroke (NDS), Disabling Stroke (DS), and Death - following an index ischaemic stroke over a 20-year time horizon. Separate transition probabilities, costs, and utilities were assigned for patients receiving ABC_Stroke_ pathway -aligned versus non-ABC care. Annual transitions include stroke recurrence (with or without functional decline) and mortality.

Model inputs, including probability values, cost parameters, and utility weights, are defined in **Table 1**.

NDS – Non-disabling stroke (mRS 0–2)

DS – Disabling stroke (mRS 3–5)

Dead – Absorbing state (no further transitions)

pABC_… / pUC_… – Transition probabilities for ABC-adherent and usual care arms

c… – Cost variables, e.g., cIndexNDS_ABC = initial cost for NDS in ABC group

u… – Utility variables, e.g., uDS = utility for DS health state

### **Supplemental Figure 2.** Impact of a 100-fold increase in ABC_Stroke_ pathway implementation costs on ICER.

ICER – incremental cost-effectiveness ratio.

**Supplementary Figure 3A.** Incremental cost-effectiveness scatterplot at a WTP of £25,000 per QALY. Green dots indicate cost-effective simulations; red dots indicate non–cost-effective simulations.

Each point represents one of 10,000 Monte Carlo simulations. Green points denote simulations where full ABC_Stroke_ adherence is cost-effective, red points where it is not. The majority of simulations fall below the WTP line, supporting cost-effectiveness of full pathway adherence.

**Supplementary Figure 3A.** Incremental cost-effectiveness scatterplot at a WTP of £15,000 per QALY. Green dots indicate cost-effective simulations; red dots indicate non–cost-effective simulations.

Each point represents one of 10,000 Monte Carlo simulations. Green points denote simulations where full ABC_Stroke_ adherence is cost-effective, red points where it is not. The majority of simulations fall below the WTP line, supporting cost-effectiveness of full pathway adherence

**Supplementary Figure 4.** Net monetary benefit distributions at a WTP of £25,000 per QALY, demonstrating higher mean benefit and narrower prediction interval for ABCStroke pathway adherence.

The NMB distribution is shifted to the right for full ABC_Stroke_ adherence (mean £106,862) compared with no components adherence (mean £69,314).

**Supplementary Figure 5.** Net monetary benefit distributions for all ABC_Stroke_ pathway components established versus any single component established at a WTP threshold of £25,000 per QALY.

The NMB distribution is shifted to the right for full ABCStroke adherence (mean £104,327) compared with single component adherence (mean £101,321).

**Supplementary Figure 6.** Incremental cost-effectiveness scatterplot comparing all ABC_Stroke_ pathway components established versus any single component established at a willingness-to-pay threshold of £25,000 per QALY.

Each point represents one of 10,000 Monte Carlo simulations. Green points denote simulations where full ABCStroke adherence is cost-effective, red points where it is not. The majority of simulations fall below the WTP line, supporting cost-effectiveness of full pathway adherence.

### **Supplemental Table 1**. Weighted Utility Calculation

#### a. mRS 0–2 Group (Non-disabling Stroke)

| mRS | Proportion | Utility | Weighted Utility |
| --- | --- | --- | --- |
| 0 | 0.2228 | 0.936 | 0.2086 |
| 1 | 0.4762 | 0.817 | 0.3893 |
| 2 | 0.3009 | 0.681 | 0.2047 |
|  |  |  | Total = 0.803 |

#### b. mRS 3–5 Group (Disabling Stroke)

| mRS | Proportion | Utility | Weighted Utility |
| --- | --- | --- | --- |
| 3 | 0.6880 | 0.558 | 0.3840 |
| 4 | 0.2640 | 0.265 | 0.0700 |
| 5 | 0.0470 | –0.054 | –0.0022 |
|  |  |  | Total = 0.452 |

Utilities were derived from Rivero-Arias et al. (2010) using EQ-5D values by mRS category. Proportions reflect the distribution of patients within each mRS level for the NDS (mRS 0–2) and DS (mRS 3–5) groups. Weighted averages (0.803 for NDS, 0.452 for DS) were used as utility inputs in the model.

mRS – modified Rankin Scale; NDS – Non-disabling stroke (mRS 0–2); DS – Disabling stroke (mRS 3–5); EQ-5D – EuroQol five-dimension scale.

### **Supplemental Table 2A**. Weighted Acute Cost Calculation – mRS 0–2 Group

| mRS | Proportion | Acute Cost (£) | Weighted Cost (£) |
| --- | --- | --- | --- |
| 0 | 0.2228 | 3,145 | 700.44 |
| 1 | 0.4762 | 3,700 | 1,762.03 |
| 2 | 0.3009 | 4,255 | 1,280.81 |
|  |  | Total | 3,743.28 |

### **Supplemental Table 2B**. Weighted Acute Cost Calculation – mRS 3–5 Group

| mRS | Proportion | Acute Cost (£) | Weighted Cost (£) |
| --- | --- | --- | --- |
| 3 | 0.6880 | 16,409 | 11,291.39 |
| 4 | 0.2640 | 22,200 | 5,860.80 |
| 5 | 0.0470 | 26,367 | 1,254.73 |
|  |  | Total | 18,406.92 |

### **Supplemental Table 2C**. Weighted Annual Cost Calculation – mRS 0–2 Group

| mRS | Proportion | Annual Cost (£) | Weighted Cost (£) |
| --- | --- | --- | --- |
| 0 | 0.2228 | 2,846 | 634.10 |
| 1 | 0.4762 | 3,348 | 1,594.88 |
| 2 | 0.3009 | 3,850 | 1,158.17 |
|  |  | Total | 3,387.15 |

### **Supplemental Table 2D**. Weighted Annual Cost Calculation – mRS 3–5 Group

| mRS | Proportion | Annual Cost (£) | Weighted Cost (£) |
| --- | --- | --- | --- |
| 3 | 0.6880 | 13,697 | 9,430.74 |
| 4 | 0.2640 | 18,532 | 4,893.45 |
| 5 | 0.0470 | 30,093 | 1,420.77 |
|  |  | Total | 15,744.96 |

Acute and annual costs by mRS category were taken from Luengo-Fernandez et al. (2013) for UK OXVASC registry atrial fibrillation patients after stroke. Proportions show the distribution of patients within each mRS level for the non-disabling stroke (NDS; mRS 0–2) and disabling stroke (DS; mRS 3–5) groups. Weighted costs were calculated by multiplying category-specific costs by the corresponding proportion.

mRS – modified Rankin Scale.

### **Supplemental Table 3A**. Transition Probabilities: Patients with Full ABC Stroke Pathway Care (No Disability at Baseline, n=104)

| From / To | No Recurrent Stroke | Recurrent NDS | Recurrent DS | Death |
| --- | --- | --- | --- | --- |
| Index Stroke only | 0.96153846 | 0.02884615 | 0.00961538 | 0 |
| Recurrent Non-Disabling Stroke | - | 1 | - | 0 |
| Recurrent Disabling Stroke | - | - | 1 | 0 |
| Death | - | - | - | 1 |

NDS = Non-disabling stroke (mRS 0–2), DS = Disabling stroke (mRS 3–5).

**Supplemental Table 3B.** Transition Probabilities (Small-Sample Adjusted): Patients with Full ABC Stroke Pathway Care (No Disability at Baseline, *n* = 104).

| From / To | No Recurrent Stroke | Recurrent NDS | Recurrent DS | Death |
| --- | --- | --- | --- | --- |
| Index Stroke only | 0.96153846 | 0.02884615 | 0.00961538 | 0 |
| Recurrent Non-Disabling Stroke | - | 0.719246959775491 (SE 0.171799704201482)* | - | 1-0.719246959775491 (SE 0.171799704201482)* |
| Recurrent Disabling Stroke | - | - | 0.603271645736946 (SE 0.222335404035332)* | 1-0.603271645736946 (SE 0.222335404035332)* |
| Death | - | - | - | 1 |

Values marked with an asterisk (*) were adjusted using the Agresti–Coull and Wilson methods at the 95% confidence level (*z* = 1.96).

NDS = Non-disabling stroke (mRS 0–2), DS = Disabling stroke (mRS 3–5).

### **Supplemental Table 4A**. Transition Probabilities: Patients With Full ABC_Stroke_ pathway Care (With Disability at Baseline, n=51)

| From / To | No Recurrent Stroke | Recurrent DS | Death |
| --- | --- | --- | --- |
| No Recurrent Stroke | 0.92307692 | 0.05769231 | 0.01923077 |
| Recurrent Disabling Stroke | - | 1 | 0 |
| Death | - | - | 1 |

### DS = Disabling stroke (mRS 3–5). Patients do not transition back to NDS once DS has occurred.

### **Supplemental Table 4B.** Transition Probabilities (Small-Sample Adjusted): Patients with Full ABC Stroke Pathway Care (With Disability at Baseline, *n* = 51).

| From / To | No Recurrent Stroke | Recurrent DS | Death |
| --- | --- | --- | --- |
| No Recurrent Stroke | 0.92307692 | 0.05769231 | 0.01923077 |
| Recurrent Disabling Stroke | - | 0.719246959775491 (SE 0.171799704201482)* | 1-0.719246959775491 (SE 0.171799704201482)* |
| Death | - | - | 1 |

### Values marked with an asterisk (*) were adjusted using the Agresti–Coull and Wilson methods at the 95% confidence level (*z* = 1.96).

### DS = Disabling stroke (mRS 3–5). Patients do not transition back to NDS once DS has occurred.

### **Supplemental Table 5A**. Transition Probabilities: Patients Without Any ABC Pathway Care (No Disability at Baseline, n=113)

| From / To | No Recurrent Stroke | Recurrent NDS | Recurrent DS | Death |
| --- | --- | --- | --- | --- |
| Index Stroke only | 0.902654867 | 0.03539823 | 0.02654867 | 0.03539823 |
| Recurrent Non-Disabling Stroke | - | 1 | - | 0 |
| Recurrent Disabling Stroke | - | - | 0.33333333 | 0.66666667 |
| Death | - | - | - | 1 |

NDS = Non-disabling stroke (mRS 0–2), DS = Disabling stroke (mRS 3–5).

**Supplemental Table 5B.** Transition Probabilities (Small-Sample Adjusted): Patients without Any ABC Pathway Care (No Disability at Baseline, n = 113).

| From / To | No Recurrent Stroke | Recurrent NDS | Recurrent DS | Death |
| --- | --- | --- | --- | --- |
| Index Stroke only | 0.902654867 | 0.03539823 | 0.02654867 | 0.03539823 |
| Recurrent Non-Disabling Stroke | - | 0.755049989798 (SE 0.15357631454396)* | - | 1 - 0.755049989798 (SE 0.15357631454396) |
| Recurrent Disabling Stroke | - | - | 0.426917680074836 (SE 0.189104444411128)* | 1-0.426917680074836 (SE 0.189104444411128)* |
| Death | - | - | - | 1 |

Values marked with an asterisk (*) were adjusted using the Agresti–Coull and Wilson methods at the 95% confidence level (z = 1.96).

NDS = Non-disabling stroke (mRS 0–2), DS = Disabling stroke (mRS 3–5).

### **Supplemental Table 6A**. Transition Probabilities: Patients Without Any ABC_Stroke_ pathway Care (With Disability at Baseline, n=39)

| From / To | No Recurrent Stroke | Recurrent DS | Death |
| --- | --- | --- | --- |
| No Recurrent Stroke | 0.61538462 | 0.12820513 | 0.25641026 |
| Recurrent Disabling Stroke | - | 0.2 | 0.8 |
| Death | - | - | 1 |

DS = Disabling stroke (mRS 3–5). Once patients have еxperienced DS, recovery to NDS is not modelled.

**Supplemental Table 6B.** Transition Probabilities (Small-Sample Adjusted): Patients without Any ABC Stroke Pathway Care (With Disability at Baseline, n = 39).

| From / To | No Recurrent Stroke | Recurrent DS | Death |
| --- | --- | --- | --- |
| No Recurrent Stroke | 0.61538462 | 0.12820513 | 0.25641026 |
| Recurrent Disabling Stroke | - | 0.330347448425624  (SE 0.158177537694477)* | 1- 0.330347448425624  (SE 0.158177537694477)* |
| Death | - | - | 1 |

Values marked with an asterisk (*) were adjusted using the Agresti–Coull and Wilson methods at the 95% confidence level (z = 1.96).

DS = Disabling stroke (mRS 3–5). Once patients have еxperienced DS, recovery to NDS is not modelled.

### **Supplemental Table 7**. ABC_Stroke_ pathway Delivery Costs

Weighted costs were calculated based on the prevalence of comorbidities and clinical indications for each ABC component observed in the Athens cohort.

#### a. Weighted Annual Costs (recurring every year)

| Component | Cost (£) |
| --- | --- |
| Anticoagulants (21.8% of patients) | £85.26 |
| Antihypertensive therapy (85.3%) | £39.24 |
| Statins (100%) | £16.33 |
| Diabetes treatment (12.8%) | £10.18 |
| BP self-monitoring (100%) | £57.35 |
| NHS Health Check (100%) | £30.90 |
| AFib pulse palpation (3× annually) | £1.80 |
| Total Annual Cost | £241.04 |

#### b. Non-weighted Full NHS Pricing (Annual Components)

| Component | Full Cost (£) |
| --- | --- |
| Anticoagulants | £391.00 |
| Antihypertensive therapy | £46.00 |
| Statins | £16.33 |
| Diabetes treatment | £79.59 |
| BP self-monitoring | £57.35 |
| NHS Health Check | £30.90 |
| Atrial fibrillation pulse palpation (3× annually) | £1.80 |

#### c. Weighted One-Off Costs (delivered once per patient)

| Component | Cost (£) |
| --- | --- |
| Pharmacy/medical/medicine services (100%) | £28.00 |
| Diabetes education (12.8%) | £11.39 |
| Weight management (64%) | £33.92 |
| Smoking cessation (40.4%) | £59.79 |
| Total Initial Cost | £133.10 |

#### d. Non-weighted Full NHS Pricing (One-Off Components)

| Component | Full Cost (£) |
| --- | --- |
| Pharmacy services | £28.00 |
| Diabetes education | £89.00 |
| Weight management | £53.00 |
| Smoking cessation | £148.00 |

#### e. Summary of ABC Pathway Costs

| Cost Type | Amount (£) |
| --- | --- |
| Annual Costs | £241.04 |
| Initial Costs | £133.10 |

**Supplemental Table 8.** Transition Probabilities (Small-Sample Adjusted): Patients with Only One Component of the ABC Pathway Established (No Disability at Baseline, n = 660).

| From / To | No Recurrent Stroke | Recurrent NDS | Recurrent DS | Death |
| --- | --- | --- | --- | --- |
| Index Stroke only | 0.89090909 | 0.05606061 | 0.02121212 | 0.03181818 |
| Recurrent Non-Disabling Stroke | - | 0.952969521272428  (SE 0.15357631454396)* | - | 1 - 0.952969521272428  (SE 0.15357631454396)* |
| Recurrent Disabling Stroke | - | - | 0.5 (SE 0.11837312285982)* | 1- 0.5 (SE 0.11837312285982)* |
| Death | - | - | - | 1 |

Values marked with an asterisk (*) were adjusted using the Agresti–Coull and Wilson methods at the 95% confidence level (z = 1.96).

NDS = Non-disabling stroke (mRS 0–2), DS = Disabling stroke (mRS 3–5).

**Supplemental Table 9.** Transition Probabilities (Small-Sample Adjusted): Patients with Only One Component of the ABC Stroke Pathway Care Established (With Disability at Baseline, *n* = 176).

| From / To | No Recurrent Stroke | Recurrent DS | Death |
| --- | --- | --- | --- |
| No Recurrent Stroke | 0.66666667 | 0.09090909 | 0.24242424 |
| Recurrent Disabling Stroke | - | 0.479872471982481  (SE 0.100236999085976 )* | 1- 0.479872471982481  (SE 0.100236999085976 )* |
| Death | - | - | 1 |

Values marked with an asterisk (*) were adjusted using the Agresti–Coull and Wilson methods at the 95% confidence level (*z* = 1.96).

DS = Disabling stroke (mRS 3–5). Once patients have еxperienced DS, recovery to NDS is not modelled.

## **Supplemental Table 10.** Baseline Characteristics of Patients in the ATHENS Registry According to Adherence to the ABC Stroke Pathway

| **Characteristic** | **0 Components** | **1 Component** | **3 Components** |
| --- | --- | --- | --- |
| **n** | **192** | **956** | **156** |
| Year of admission, median (IQR) | 2002 (1994–2007) | 2000 (1995–2007) | 2008 (2006–2010) |
| Female, n (%) | 96 (50.0) | 361 (37.8) | 56 (35.9) |
| Age, median (IQR) | 75 (67–83) | 72 (63–80) | 68 (59–75) |
| NIHSS, median (IQR) | 5 (2–21) | 4 (2–10) | 6 (2–13) |
| Obesity, n (%) | 50 (26.2) | 289 (30.3) | 100 (64.1) |
| Hypertension, n (%) | 131 (68.2) | 682 (71.3) | 133 (85.3) |
| Atrial fibrillation, n (%) | 138 (71.9) | 372 (38.9) | 34 (21.8) |
| Diagnosed on admission | 17 (8.9) | 24 (2.5) | 4 (2.6) |
| Diagnosed during hospitalisation | 22 (11.5) | 63 (7.0) | 5 (3.2) |
| Diagnosed on follow-up | 12 (6.3) | 32 (3.4) | 2 (1.3) |
| Known atrial fibrillation | 87 (45.3) | 253 (26.5) | 23 (14.7) |
| OACs prior to stroke, n (%) | 10 (5.2) | 55 (5.8) | 16 (10.3) |
| AF treated with OACs prior to stroke | 7 (3.7) | 45 (4.7) | 13 (8.3) |
| Antiplatelet prior to stroke, n (%) | 33 (17.2) | 182 (19.0) | 32 (20.5) |
| Diabetes mellitus, n (%) | 43 (22.4) | 268 (28.0) | 20 (12.8) |
| Hyperlipidaemia, n (%) | 50 (26.0) | 292 (30.5) | 101 (64.7) |
| Coronary artery disease, n (%) | 28 (14.6) | 200 (20.9) | 34 (21.8) |
| Peripheral artery disease, n (%) | 6 (3.1) | 41 (4.3) | 4 (2.6) |
| Prior stroke, n (%) | 13 (6.8) | 54 (5.6) | 15 (9.6) |
| Heart failure, n (%) | 20 (10.4) | 73 (7.6) | 7 (4.5) |
| Prosthetic valve, n (%) | 4 (2.1) | 9 (0.9) | 1 (0.6) |
| Smoking, n (%) | 47 (25.5) | 307 (32.1) | 63 (40.4) |
| Alcohol, n (%) | 7 (3.7) | 78 (8.2) | 20 (12.8) |
| Antiplatelet at discharge, n (%) | 91 (47.4) | 703 (73.5) | 125 (80.1) |
| Anticoagulation at discharge, n (%) | 18 (9.4) | 160 (16.7) | 41 (26.3) |
| Antihypertensive at discharge, n (%) | 68 (35.4) | 395 (41.3) | 133 (85.3) |
| Antidiabetic at discharge, n (%) | 5 (2.6) | 38 (4.0) | 20 (12.8) |
| Statin at discharge, n (%) | 8 (4.2) | 100 (10.5) | 156 (100.0) |
| Endarterectomy, n (%) | 2 (1.0) | 17 (1.8) | 3 (1.9) |
| Diuretics at discharge, n (%) | 38 (19.8) | 197 (20.6) | 62 (39.7) |
| Beta-blockers at discharge, n (%) | 10 (5.2) | 82 (8.6) | 40 (25.6) |
| ACE/ARB at discharge, n (%) | 38 (19.8) | 298 (24.9) | 113 (72.4) |
| Physiotherapy, n (%) | 0 | 347 (36.3) | 112 (71.8) |
| mRS at discharge, median (IQR) | 1 (1–5) | 1 (1–4) | 3 (0–4) |
| mRS at 3 months, median (IQR) | 1 (1–5) | 1 (1–3) | 1 (0–3) |

IQR = interquartile range; NIHSS = National Institutes of Health Stroke Scale; OAC = oral anticoagulant; ACE = angiotensin-converting enzyme; ARB = angiotensin receptor blocker; mRS = modified Rankin Scale.
